# Interoceptive differences in elite sprint and long-distance runners: A multidimensional investigation

**DOI:** 10.1371/journal.pone.0278067

**Published:** 2023-01-25

**Authors:** Tom Seabury, David Benton, Hayley A. Young

**Affiliations:** Swansea University, Wales, United Kingdom; University of Rome, ITALY

## Abstract

Interoception, the process of detecting and interpreting bodily sensations, may facilitate self-regulation and thereby play a crucial role in achieving elite performance in competitive sports. However, there is a lack of research conducted in world-class athletes. In the present research, two studies examined self-reported (interoceptive sensibility) and behavioural (interoceptive accuracy) interoception in elite (top 100 ranking) sprint and long-distance runners, and non-athletes. Study 1 used the Multidimensional Assessment of Interoceptive Awareness Questionnaire. Sprinters reported having better regulation of attention to internal sensations, greater emotional awareness, better self-regulation, and reported a greater propensity to listen to their body for insight, than distance runners. Compared to non-athletes, sprinters and distance runners had more bodily trust, attention regulation, and self-regualtion. Additionally, elite athletes reported lower emotional awareness, self-regulation, and body listening. Study 2 examined cardioception using two tasks: The Heartbeat Counting Task, and The Heartbeat Detection Task. Elite and non-elite runners performed the tasks under two conditions; in silence, and whilst listening to pre-recorded crowd noise that simulated the live sounds of spectators during a sporting event. Sprinters and distance runners were able to maintain heartbeat detection accuracy when distracted, whereas non-athletes could not. Across both tasks, compared to non-athletes, sprinters and distance runners were more confident than non-athletes in their interoceptive percept. Additionally, elite athletes compared to non-elite athletes were less accurate when counting their heartbeat and were characterised by a higher interoceptive prediction error. Athletic populations have altered interoceptive abilities.

## Introduction

Interoception describes the detection and perception of stimuli originating from within the body [1]. To perform well, elite track athletes must monitor and utilise internal afferent sensations, such as those related to effort and fatigue. For example, early research found that elite long-distance runners reported regulating their pace by "reading their bodies", as well as attending closely to bodily input such as "respiration" and "sensations in their feet and legs" [2]. Conversely, less elite runners reported predominantly directing their attention away from the body [2]. Unfortunately, most of the early research connecting interoception and sports performance was based solely on the athlete’s qualitative reports. However, in recent years the definition of interoception has expanded considerably. Despite once being considered a unitary concept, interoception is now understood to comprise multiple dimensions including both self-reported and behavioural components [3]. Therefore, more research is needed to determine which aspects of interoception are associated with achieving world-class performance.

Recently, several interoceptive taxonomies were proposed [1, 3–7]. Table 1 describes the interoceptive indices most commonly used in empirical research and those which were used here [1, 3–8]. Evidence indicates that some interoceptive components (i.e., accuracy, awareness, and sensibility) are empirically dissociable [3, 9, 10] and may differentially associate with behaviour [10, 11]. Consequently, when considering the role of interoception in elite sports performance, it is important that these dimensions are studied separately.

**Table 1:** Interoceptive taxonomy.

Afferent signal	Variation in signal strength / concentration of one or more afferent signals such as an increase in heart rate.
Interoceptive accuracy	One’s ability to correctly monitor internal changes, measured using objective task performance [3]. For example, Heartbeat Detection Task [12] or Heartbeat Counting Task [13] performance.
Interoceptive sensibility	One’s self-reported domain general tendency to focus on internal sensations, operationalised using a range of questionnaires [1, 3–5] Originally described by Garfinkel, Seth [3] as a unitary phenomenon, Mehling [5] argued that interoceptive sensibility is itself multidimensional including both anxiety driven, evaluative/avoidant and mindful, non-judgmental, and accepting attentional styles. Accordingly, the Multidimensional Assessment of Interoceptive Awareness (MAIA), which assesses 8-items via self-report (Noticing, Not-Distracting, Not Worrying, Attention Regulation, Emotional Awareness, Self-Regulation, Body Listening, and Trusting) became one of the most widely used interoception [14].
Interoceptive confidence	One’s postdictive confidence in their ability to accurately monitor internal changes associated with a particular interoceptive task or channel [15, 16]. It was suggested that raw confidence ratings might reflect one’s subjective belief about the accuracy of their interoceptive percept within a particular domain [6].
Interoceptive awareness (or insight)	One’s metacognitive judgement regarding one’s interoceptive accuracy, assessed as the correspondence between objective accuracy and subjective confidence ratings [3]. Note that interoceptive insight has been operationalised in two ways: (i) calibration: how well confidence tracks accuracy on a trial-by-trial basis [3]; and (ii) a prediction error index (PI) capturing the magnitude of the difference between sensibility and accuracy [3].

Despite the paucity of research examining interoception in athletes, several recent theoretical reviews have been published that have emphasised the role of interoception in physical activity [17–20]. For example, Tucker [21] proposed a model whereby the regulation of exercise work rate during self-paced exercise is achieved by combining interoceptive feedback (e.g., body temperature, metabolite concentrations, arterial saturation levels, increased ventilatory rates and increased heart rates which generates conscious feelings of exertion) and existing expectations about the optimal rate of exertion required for the exercise (e.g., based on expected duration/ distance, motivations / goals which produces anticipated feelings of exertion against which the experienced exertion is compared). This idea has similarities to recent applications of prediction processing to interoception which imply that subjective feeling states are determined by predictions about the interoceptive state of the body [22, 23]. These models provide the theoretical foundations for the proposed influence of interoception on self-regulation during exercise. However, hitherto empirical verification of these models has been limited.

Nonetheless, there is some experimental evidence that better interoception might facilitate self-regulation during exercise [24–26]. For example, Herbert, Ulbrich [25] conducted one of the first studies on heartbeat perception accuracy and exercise performance. Using a bike ergometer, participants were instructed to set their own pace for fifteen minutes. Those who were more accurate at counting their heartbeat had lower cardiac output and lower heart rate during the exercise. In addition, they cycled a shorter distance in the time limit. Herbert, Ulbrich [25] interpreted these findings as suggesting that poor heartbeat perceivers may be less efficient at regulating their pace during physical effort. This interpretation is consistent with more recent evidence indicating that better heartbeat perceivers were better able to replicate their physical effort, albeit only at lower training intensities [27]. If better interoception does facilitate self-regulation during exercise, this would have clear implications for long-distance athletes where optimal ‘pacing’ is crucial to achieving high level performance. Nonetheless, an alternative explanation for Herbert, Ulbrich [25] findings is that those with better interoception had more difficulties performing physical activity at the limit of their physical abilities. For example, those with higher metacognitive awareness of their interoceptive accuracy reported more fatigue during a knee extension endurance task after having been pre-fatigued [26]. Therefore, it is also plausible that athletes who are particularly sensitive to internal sensation may struggle to push beyond their physiological boundaries as required in elite athletes. In addition, this could be particularly important in sprint athletes who are required to train at high intensities. Supporting this suggestion, interventions that reduce interoceptive attention may lower the rate of perceived excursion associated with physical exertion [28].

Interestingly, the results of one study could help clarify these alternative interpretations [11]. It was reported that the association between the ability to perceive one’s heartbeat and the behavioural regulation of exercise performance might depend on one’s subjective response to the perturbation of internal signals [11]. Those who were more accurate at perceiving their heartbeat decreased their power output during a 30-second Wingate sprint task, but only if they reported high levels of subjective anxiety sensitivity [11]. These findings illustrated how individual dispositional tendencies may alter inferences made about interoceptive perturbations, thereby dictating whether better interoceptive abilities facilitate or debilitate physical performance. In addition, they further highlight the importance of understanding the possible inter-relationships between key interoceptive concepts, regarding physical activity.

Although the supposed links between interoception and physical activity are not new, limited research has compared athletes and non-athletes. Faull, Cox [29] compared the ventilatory awareness of endurance athletes (defined as training five or more times per week in cycling, rowing, or distance running) and non-athletes. Participants completed a hypercapnic ventilatory response test during which they self-reported their breathlessness anxiety and intensity. The hypercapnic ventilatory response did not differ between athletes and non-athletes indicating that central chemoreceptor sensitivity was similar in both populations. However, the correlation between perturbated ventilation rate and breathlessness intensity and anxiety was significant only in the athlete group. This suggested that athletes were more aware of changes in their ventilation rate. Whilst this increased awareness could benefit self-regulation during physical exertion, the emotional component of breathlessness anxiety could also be a limiting factor. It is important to note that the domain generality of interoceptive signals is currently debated [30], and these effects may be limited to ventilatory interoception. In addition, it is unclear whether these effects vary according to the athlete’s sport.

**Fig 1:**
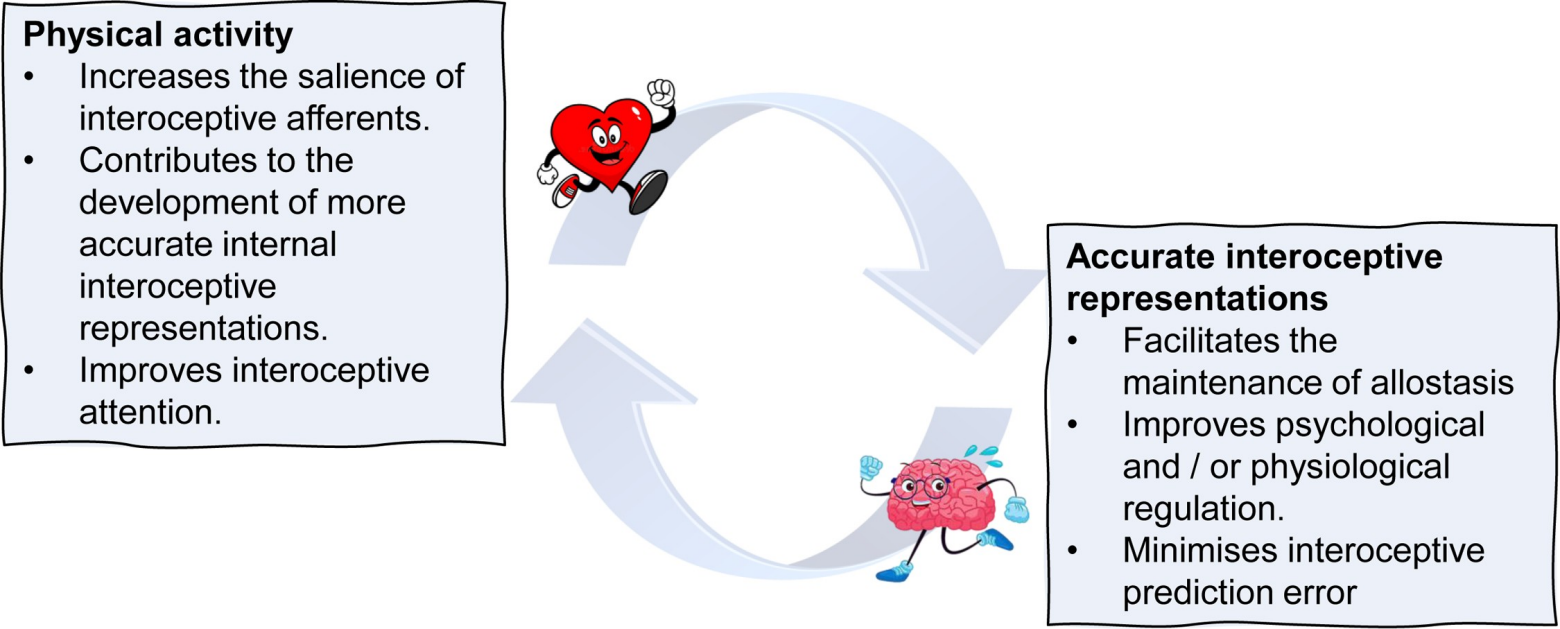
Positive feedback loop between physical activity and interoception. Note. This model shows the reciprocal association between acute and chronic physical activity and interoceptive processes. Physical activity increasing the salience of interoceptive signals enhancing interoceptive accuracy and attention. Accurate internal models facilitate allostasis and minimise prediction error during subsequent exertion.

Hitherto, studies comparing interoception across different sports are rare and have resulted in inconsistent findings. For example, Jones and Hollandsworth [31] compared the performance of those who were sedentary, intermediate tennis (which requires short burst of anaerobic activity over a prolonged period of time) players, and distance runners (mostly aerobic) on a heartbeat detection task, both before and after exercise that raised resting heartrate by 75%. Male runners were more accurate at rest, and exercise increased the performance of other groups to a similar level. Notably, the increased interoceptive performance observed after exercise suggested that an increased salience of cardiac afferents during and after acute physical activity could enhance interoceptive abilities. Taken together with the previously mentioned studies, this indicates a potential reciprocal association between physical activity and interoceptive processes [17]. Specifically, physical activity increases the salience of interoceptive afferents thereby enhancing the development of accurate internal interoceptive models. These models then contribute to the maintenance of allostasis during subsequent physical exertion (Fig 1).

Interestingly, Hirao, Vogt [32] hypothesised that long-distance runners would have better interoception than sprinters. Interoceptive accuracy (using the heartbeat counting task), and sensibility (using the Multidimensional Assessment of Interoceptive Awareness questionnaire (MAIA) [14]), were examined in university-level athletes who competed in either event. Contrary to their hypothesis, Hirao, Vogt [32] found that sprinters scored significantly higher in subjective attention regulation (belief in their ‘ability to sustain and control attention to body sensations’) than their endurance counterparts. No differences were found in interoceptive accuracy.

There are several possible explanations for these inconsistent results. Firstly, a limitation of Hirao, Vogt [32] was that they used the heartbeat counting paradigm to measure interoceptive accuracy. This is a concern because there are often methodological differences in how the heartbeat counting task is conducted which might explain mixed results from recent meta-analysis [33, 34]. The heartbeat counting paradigm is also potentially biased by non-interoceptive factors, such as previous knowledge of resting heartrate [35]. This might be a particularly relevant consideration when testing athletes, given that athletes are often exposed to technology such as heart rate monitors and smartwatches. In this context, heartbeat detection tasks, which are not influenced by explicit knowledge of heartrate [35], might offer a more rigorous assessment of interoceptive accuracy.

Secondly, Jones and Hollandsworth [31] combined interoceptive certainty (i.e., confidence ratings) and heartbeat detection performance into a single ‘accuracy’ score. It is now recognised that these represent empirically dissociable interoceptive dimensions [3]. Finally, prior research has not considered that effects could vary according to the athlete’s level of expertise. Indeed, answering this question will be essential if interventions targeting interoception are to be recommended for enhancing sports performance.

In summary, there is good theoretical and some empirical evidence that interoception (accuracy and/ or awareness) might be related to self-regulation during exercise [17–20, 25–27]. In addition, athletes may have better interoceptive abilities than non-athletes [29, 31]. However, conclusions are so far limited due to studies taking a unidimensional approach [25], methodological inconsistencies [31, 32]’, and the recruitment of college-level, rather than elite athletes [29, 31, 32]. The present studies aimed to address some of these concerns by taking a multidimensional approach to understanding interoception in world-class (ranked in the top 100) sprint and long-distance athletes, and non-athletes. These populations were chosen to extend the work by Hirao, Vogt [32]. Two questions were addressed: (1) whether sprinters, distance runners and non-athletes differ in their interoceptive abilities, and (2) whether elite athletes differ from non-elite athletes in their interoceptive abilities. In a large online sample, study one considered whether elite and non-elite athletes could be differentiated according to their self-reported interoceptive attentional and regulatory styles. Study two used both a counting task and a ‘gold standard’ Heartbeat Detection Task to examine differences in interoceptive accuracy, confidence, and metacognitive awareness. Although previous research has considered interoception in either the respiratory or the cardiac domain, here we focused on cardioception for consistency with the study by Hirao, Vogt [32]. In both studies we had three hypotheses: (1) that athletes would show better interoceptive abilities than non-athletes (directional); (2) that elite athletes would differ from novices in their interoception (non-directional); and (3) that interoception would vary based on the athletes’ sport (non-directional).

## Study 1

### Method

#### Participants

213 participants (141 males) aged between 18 and 56 (M = 24.99, SD = 7.541) participated in this study (Table 1). The sample consisted of 50 sprinters (100m, 200m and 400m; 39 males) and 67 distance runners (800–10,000m; 49 males) as well as 96 non-athlete controls (53 males). Concerning rank, the athlete sample consisted of 70 elite athletes (top 100 national ranking; 50 males) and 47 non-elite athletes (outside top 100 national ranking; 38 males). This ranking was based on best career rank achieved within their discipline. Groups were well matched in terms of age (Table 2). Sprinters and distance runners were well matched on gender (X^2^ = 0.536, p = 0.464). However, the non-athlete group had proportionately higher number of females (X^2^ = 10.197, p<0.006). Gender did not vary according to rank (X^2^ = 1.622, p<0.145). Regarding level of education, all participants were educated to at least undergraduate degree level. Two participants (one athlete and one non-athlete) were removed from the sample due to being over retirement aged (over 65) so considered past competitive sporting age. Power analysis was not conducted due to the niche sample, and because there were no previous effect sizes that used elite athletes. Participants were not compensated for their time.

**Table 2:** Demographics and sample means of athletes in Study 1.

	Elite		Non-Elite		Non-athletes	TOTAL
	Sprinters	Distance Runners	Sprinters	Distance Runners		
**N**	32	38	18	29	96	213
**Age**	23.61 (6.77)	25.21(10.40)	22.00 (7.24)	25.31(10.42)	26.57 (6.04)	24.99 (7.54)
**Noticing**	16.32 (4.46)	16.92 (3.22)	17.82 (3.34)	16.96 (3.87)	16.30 (4.24)	16.90 (3.74)
**Anxiety**	3.34 (1.49)	3.38 (1.90)	3.17 (1.89)	3.21 (1.82)	3.77 (1.82)	3.51 (1.80)
**Depression**	2.50 (1.52)	2.90 (2.07)	2.83 (2.12)	2.45 (1.88)	2.95 (1.68)	2.79 (1.80)
**Attention Regulation**	29.19 (6.93)	26.47 (6.23)	32.30 (6.85)	28.43 (6.84)	25.69 (7.05)	28.57 (6.86)
**Emotional awareness**	20.77 (5.02)	19.53 (4.84)	24.29 (3.60)	21.57 (5.54)	22.87 (6.24)	21.05 (5.08)
**Self-regulation**	15.42 (4.50)	14.95 (3.68)	18.76 (3.35)	16.09 (4.38)	14.04 (4.70)	15.92 (4.18)
**Body Listening**	9.23 (3.94)	8.37 (3.21)	11.88 (3.55)	9.87 (3.75)	8.66 (3.58)	9.48 (3.74)
**Trusting**	13.29 (3.49)	13.92 (3.15)	14.82 (2.53)	14.30 (2.85)	11.77 (3.86)	13.96 (3.11)

*Note*. Data are mean (SD). There were no differences in rating of depression [EVENT (F(1,209) = 0.00, p = .906), RANK (F(1, 209) = 0.28, p = .867), ATHLETE(F(1, 209) = 1.21, p = .271)]. Anxiety did not vary according to EVENT (F(1,209) = 0.14, p = .906) or RANK (F(1,209) = 0.26, p = .607). However, athletes were less anxious than non-athletes (F(1,209) = 3.87, p = .050).

#### Procedure

Participants were recruited through various platforms, including word of mouth, team emails, social media forums, and groups (e.g., Facebook). Elite athletes were accessed either directly through these methods, or through initial contact with their coaches. Non-athlete controls were recruited from the Swansea University Psychology Participant Pool, as well as word of mouth. The participants accessed and completed the questionnaire online (Qualtrics, Provo, UT) by either following a hyperlink button or through a manufactured QR code linking to the study. Participants with a BMI > 30kg/m^2^ were excluded, as were smokers, those with any metabolic or cardiovascular disorder, gastrointestinal problems, pregnancy and a diagnosis of a mood or eating disorder or other psychological or neurological disorder. Participants provided their consent by continuing to the questionnaire after reading the information page and consent form. Participants first completed the MAIA, then provided their age, gender, ethnicity, sporting event, and best-ever sporting rank. Finally, they reported their anxiety and depression levels. This study took approximately 15 minutes to complete. At the end, participants were thanked for their time and debriefed. All procedures were approved by the Swansea University Ethics Committee.

#### Interoceptive sensibility (IS)

Interoceptive sensibility was measured using the Multidimensional Assessment of Interoceptive Awareness (MAIA) [14]. This 32-item multidimensional instrument assesses eight concepts related to interoception: (1) Noticing–"Being aware of comfortable/uncomfortable and neutrally based bodily sensations" (*α* = .732). (2) Not-distracting–"Being able to ignore or distract oneself from sensations of evident discomfort or pain" (*α* = .578). (3) Not Worrying–"Ability to either not worry or not experience emotional distress, while experiencing physical pain or discomfort" (*α* = .477). (4) Attention regulation–"Being able to sustain and control your attention to bodily sensations" (*α* = .892). (5) Emotional awareness–"Being aware of the connection between bodily sensations and emotional states" (*α* = .835). (6) Self-regulation–"Being able to regulate distress by attention to the bodily sensations" (*α* = .845). (7) Body listening–"Active listening to the body for insight" (*α* = .836). (8) Trusting–"Experiencing the body as a safe and trustworthy place" (*α* = .905). In the present study, two measures did not reach an acceptable Cronbach’s alpha score (*α* = .70; George & Mallery, 2003) and were therefore excluded: Not distracting (*α* = .578) and not worrying (*α* = .477). Items for each scale were summed to produce a total score which were used for the main analysis.

#### Anxiety and depression

Both anxiety and depression were measured using a single depressive symptom screening question. Participants were asked ‘On a scale of 1 to 8, where 1 means no anxiety/depression and 8 means extreme anxiety/depression, how would you say you feel in general?’. A single screening question has been shown as an accurate initial screening option [36].

### Statistical analysis

**Fig 2:**
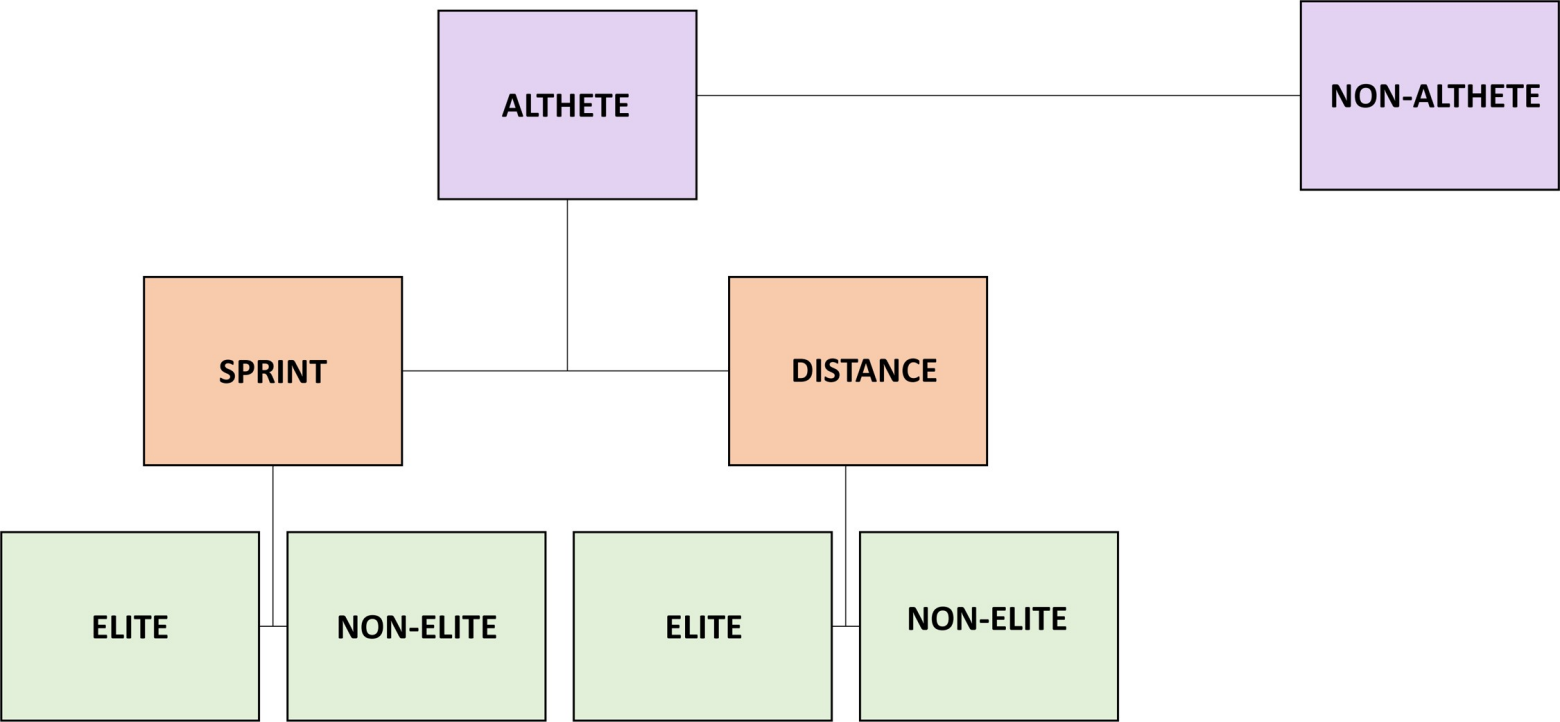
Nested design used in Study 1 and Study 2.

Data were analysed using SPSS 26 (IBM Corporation). The data was normally distributed or fulfilled the Likert scale statistical assumptions for parametric analysis [37]. As data were nested (Fig 2), a series of hierarchical ANOVAs were used. Specifically, the models examined whether interoception varied according to: (1) ATHLETE (i.e., athletes versus non-athletes), (2) EVENT (nested within ATHLETE) (i.e., sprinters versus distance athletes), (3) RANK (nested within ATHLETE) (i.e., elite (top 100) versus non-elite (not top 100) athletes), and (4) RANK X EVENT (nested within ATHLETE) (i.e., whether any effect of rank depended on the participants sport). Each MAIA subscale was analysed separately. Depression, anxiety, age, and gender were entered as covariates. Where groups i.e., ATHLETE, EVENT or RANK, differed significantly regarding interoception, *t* tests were used to determine the nature of the effect. Means (*SD*) for each group are reported in Table 2. Cook’s distance was used to detect possible outliers [38]. To avoid removing excess variability, a liberal threshold of .04 was set. Cases with a Cook’s distance exceeding this threshold were excluded and the data re-analysed. To control the proportion of type 1 errors Benjamini and Hochberg’s false discovery rate (FDR) was used. The FDR was controlled at *δ* = .05. Where significant interactions did not reach this threshold, this is indicated in the text. Confidence intervals for simple effects were adjusted using the Bonferroni correction.

### Results: Type of sport

#### Noticing

No outliers were detected. The effect of ATHLETE was not significant (F(1, 204) = 0.55, p = .458, η^2^p = .003). In addition, there was no effect of EVENT (F(1, 204) = 0.25, p = .615, η^2^p = .001). Similarly the effect of RANK (F(1, 204) = 0.18, p = .672, η^2^p = .001) was not significant, and neither was the RANK X EVENT interaction (F(1, 204) = 1.48, p = .224, η^2^p = .007).

#### Attention regulation

**Fig 3:**
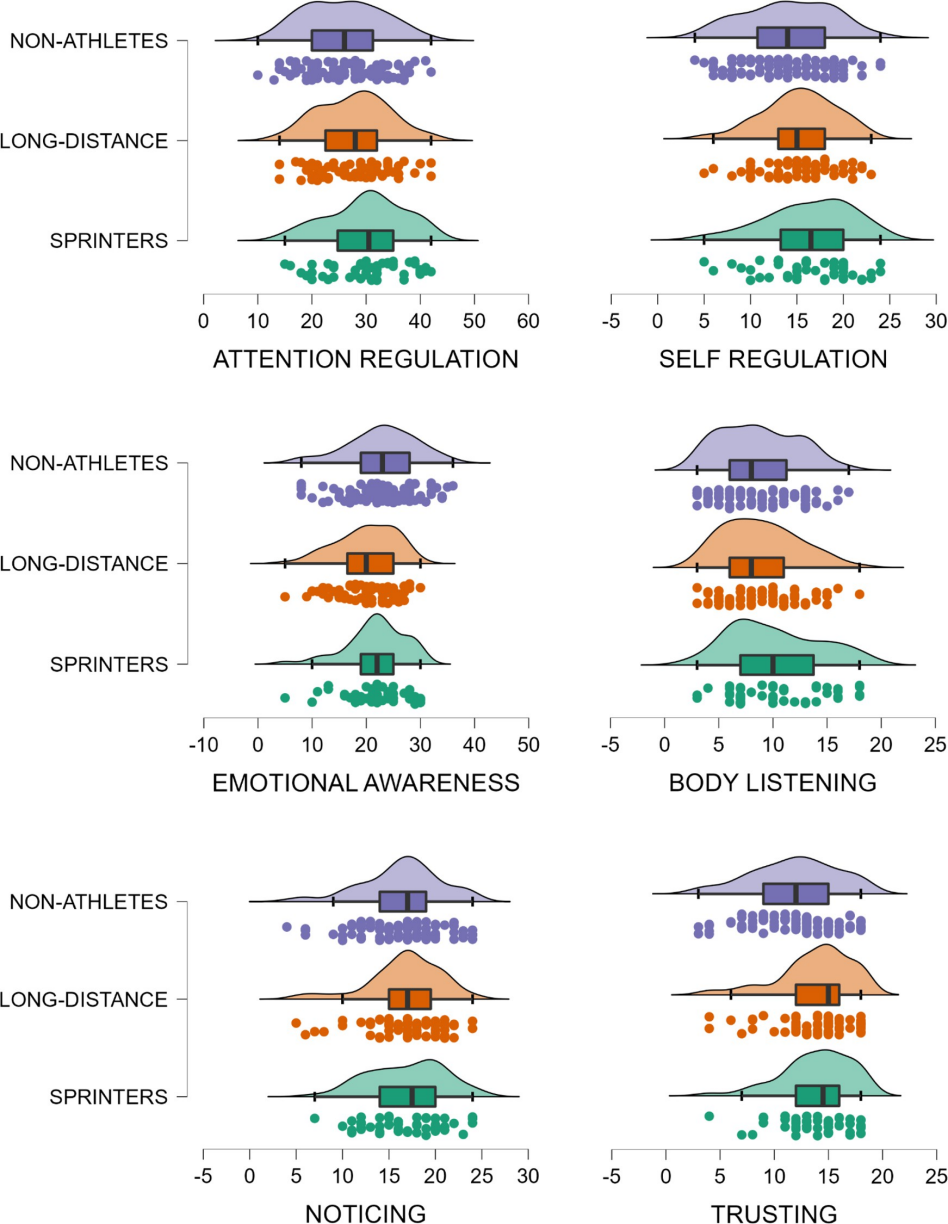
Raincloud plots showing interoceptive sensibility in sprinters, long-distance runners, and non-athletes. Note. Data are adjusted means (after adjusting for anxiety, depression, gender, and age).

Three outliers with a Cook’s distance > .04 were detected and removed. There was a main effect of ATHLETE (F(1, 201) = 9.40, p = .002, η^2^p = .045); athletes reported better attention regulation than non-athletes. There was also a significant effect of EVENT (F(1, 201) = 4.19, p = .042, η^2^p = .020); sprinters reported better attention regulation than distance athletes (Fig 3). The main effect of RANK did not reach significance (F(1, 201) = 3.03, p = .083, η^2^p = .015). Similarly, there was no EVENT X RANK interaction (F(1, 201) = 0.01, p = .902, η^2^p = .000).

#### Emotional awareness

Two outliers with a Cook’s distance > .04 were detected and removed. The main effect of ATHLETE did not reach significance (F(1, 202) = 2.83, p = .094, η^2^p = .014). The effect of EVENT was borderline indicating that sprinters reported higher emotional awareness (F(1, 202) = 3.82, p = .052, η^2^p = .019), however, the effect did not survive the FDR correction. There was a significant main effect of RANK (F(1, 202) = 4.87, p = .028, η^2^p = .024); elite athletes reported a lower level of emotional awareness. The interaction EVENT X RANK was not significant (F(1, 202) = 0.62, p = .430, η^2^p = .003).

#### Self-regulation

One outlier with a Cook’s distance > .04 was detected and removed. Athletes reported better self-regulation than non-athletes (*F*(1, 203) = 8.21, *p* = .005, η^2^p = .039). There was also an effect of EVENT (*F*(1, 203) = 3.94, *p* = .048, η^2^p = .019); sprinters reported better self-regulation than distance runners, although this effect did not survive the FDR correction. The main effect of RANK reached significance indicating that elite athletes reported lower self-regulation than non-elite athletes (*F*(1, 203) = 4.27, *p* = .040, η^2^p = .021). The EVENT X RANK interaction did not reach significance (*F*(1, 203) = 2.66, *p* = .104, η^2^p = .013).

#### Body listening

Again, one outlier with a Cook’s distance > .04 was detected and removed. The effect of ATHLETE was not significant (*F*(1, 203) = 2.23, *p* = .137, η^2^p = .011). A borderline significant effect of EVENT indicated that sprinters reported more body listening than distance runners, however the effect did not survive the FDR correction (*F*(1, 203) = 3.76, *p* = .054, η^2^p = .018). However, there was a main effect of RANK (*F*(1, 203) = 4.68, *p* = .032, η^2^p = .023); elite athletes reported lower levels of body listening than non-elite athletes. The EVENT X RANK interaction was not significant (*F*(1, 203) = 1.09, *p* = .297, η^2^p = .005).

#### Trusting

Two outliers with a Cook’s distance > .04 were detected and removed. The main effect of ATHLETE was significant (*F*(1, 202) = 15.90, *p* < .001, η^2^p = .073); athletes reported trusting their body more than non-athletes (Fig 3). The effect of EVENT was not significant (*F*(1, 202) = 0.00, *p* = .994, η^2^p = .000), and neither was the effect RANK (*F*(1, 202) = 1.22, *p* = .269, η^2^p = .006). Similarly, the EVENT X RANK interaction was not significant (*F*(1, 202) = 0.83, *p* = .361, η^2^p = .004).

## Study 2

In Study 1 self-reported interoceptive sensibility varied with both sport and the level of the athlete. However, the most important limitation of Study 1 was the use of only self-reported interoception measures. Whilst the use of questionnaires enabled the recruitment of a hard-to-reach population, it is increasingly clear that self-reported and behavioural interoception measures are empirically dissociable [10]. Therefore, it is possible that athletes only *believe* they have better interoception skills, when in fact they may not. As such, Study 2 had the aims: (I) to determine whether elite athletes differed from novices in objective interoception, (II) to establish whether effects varied depending on the athlete’s sport, and (III) to establish whether any differences were due to a better ability to regulate attention to interoceptive signals when faced with distracting conditions. Specifically Study 2 utilised the heartbeat counting task [13], and the ‘gold standard’ heartbeat detection paradigm which provides a measure of interoceptive precision that is not contaminated by explicit expectations [35]. Novices and experts performed the tasks under two conditions: (i) in silence, and (II) whilst listening to pre-recorded crowd noise that simulated the live sounds of spectators during a sporting event. The latter served as an ecologically valid distractor so that we could objectively assess athlete’s ability to sustain attention to internal signals during distraction [39].

### Method

#### Participants

In the second study, athletes residing in the local area were recruited via word of mouth, team emails, and social media groups (e.g., Facebook). This sample consisted of 58 males aged between 18 and 53 (Table 3). The sample contained 29 athletes: 14 elite (top 100 national rankings), and 15 non-elite (outside top 100 national rankings). There were 17 sprinters (100m, 200m and 400m), 12 distance runners (800–10,000m) and 29 non-athlete controls. Note that due to technical problems concerning the ECG, accuracy data were only available for n = 27 non-athletes. The non-athlete controls were Swansea University Psychology students who received course credits for their participation. Inclusion / exclusion criteria were the same as in Study 1. All participants were educated to at least undergraduate degree level. Participants refrained from drinking alcohol and physical activity within 24 h of the study, and from consuming any food and drink for at least 2 h before attending the laboratory. Testing commenced between 09.00 and 13.00. Athlete participants received no payment. The sample size was limited given the hard-to-reach population; however, it was comparable to previous research that has used track and field athletes [32, 40, 41].

**Table 3:** Demographics and sample means of athletes in Study 2.

	Elite		Non-Elite		Non-athletes	TOTAL
	Sprinters	Distance Runners	Sprinters	Distance Runners		
N	7	7	10	5	29	58
Age	21.14 (2.11)	20.86 (1.67)	22.20 (3.73)	22.40 (1.94)	24.72 (8.44)	23.19 (6.39)
HR	61.35 (9.83)	73.40 (13.95)	64.70 (6.92)	67.40 (23.60)	72.55 (15.27)	69.50 (14.52)
HBC IAc	0.70 (0.16)	0.67 (0.18)	0.87 (0.10)	0.87 (0.15)	0.72 (0.21)	0.72 (0.21)
HBC IAc–D	0.79 (0.17)	0.69 (0.15)	0.85 (0.12)	0.73 (0.10)	0.75 (0.21)	0.76 (0.18)
HBC IAw	0.07 (0.66)	-0.00 (0.85)	0.13 (0.55)	-0.14 (0.97)	-0.04 (.75)	-0.00 (0.72)
HBC IAw–D	-0.32 (0.46)	-0.12 (0.87)	0.24 (0.60)	0.02 (0.85)	-0.15 (0.67)	-0.02 (0.67)
HBC PE	1.09 (0.70)	1.35 (0.83)	0.44 (0.54)	0.68 (0.31)	0.96 (0.85)	0.91 (0.78)
HBC PE–D	0.68 (0.63)	1.26 (0.57)	0.66 (0.73)	0.96 (0.55)	0.89 (0.89)	0.88 (0.78)
HBC CON	65.51 (17.28)	63.20 (17.08)	68.59 (16.63)	69.39 (15.06)	47.09 (22.29)	56.89 (15.58)
HBC CON–D	52.01 (31.45)	67.46 (16.99)	65.97 (21.42)	63.61 (14.36)	48.19 (27.96)	55.37 (26.01)
HBD IAc	0.16 (0.31)	0.30 (0.15)	0.20 (0.23)	0.43 (0.28)	0.22 (0.25)	0.24 (0.25)
HBD IAc–D	0.27 (0.26)	0.38 (0.23)	0.21 (0.24)	0.28 (0.18)	-0.01 (0.28)	0.13 (0.30)
HBD CON	77.66 (11.61)	67.73 (22.90)	70.60 (7.87)	76.49 (6.20)	61.90 (17.12)	67.29 (16.45)
HBD CON–D	74.05 (12.59)	70.77 (14.57)	69.16 (10.50)	78.70 (10.91)	61.50 (15.92)	67.09 (15.09)
Dep	2.29 (1.25)	2.57 (1.81)	3.00 (2.50)	2 (1.22)	2.76 (1.46)	2.66 (1.65)
Anx	2.57 (1.27)	2.57 (1.40)	2.80 (1.87)	2.80 (1.48)	3.55 (1.86)	3.12 (1.73)
Ex	2.71 (1.25)	3.10 (0.88)	2.71 (0.49)	2.40 (0.55)	2.45 (1.30)	2.62 (1.11)

*Note*: Data are mean (SD). Heart rate did not vary by EVENT (*F*(1,53) = 1.78, p = .187) or RANK (*F*(1,53) = 0.05, p = 0.813). There were no differences in rating of depression [EVENT (*F*(1,53) = 0.30, p = .582), RANK (*F*(1,53) = 0.01, p = .912)], anxiety [EVENT (*F*(1,53) = 0.00, p = .999), RANK (*F*(1,53) = 0.11, p = .732)], or habitual exercise [EVENT (*F*(1,53) = 0.67, p = .417), RANK (*F*(1,53) = 0.01, p = .934)]. DEP—depression, ANX—anxiety, EX–exercise, HR–heart rate, CON–confidence, IAw–interoceptive awareness, PE–prediction error, IAc–interoceptive accuracy, D–distraction, HBC–heartbeat counting, HBD–heartbeat detection.

#### Procedure

After arriving at the laboratory and providing their written informed consent, participants were fitted with conventional Ag/AgCl electrodes and transducers which were attached using a lead III configuration: right arm (RA), left arm (LA) and left leg (LL). Once connected, the participants completed the heartbeat counting and heartbeat discrimination tasks (described below) once in silence, and again listening to crowd noise (in a counterbalanced order). Note that although distraction conditions were counterbalanced across participants, all participants completed both versions of the heartbeat counting task before completing the heartbeat detection task. As the heartbeat detection task was long participants were offered a fifteen-minute break in between conditions. As with Study 1, all procedures were approved by the Swansea University Ethics Committee and carried out in accordance with the Declaration of Helsinki (2008).

#### Measures

Interoceptive abilities were measured using two tasks: The Heartbeat Counting Task [13] and The Heartbeat Discrimination Task [35]. Interoceptive indices that were calculated (accuracy, confidence, awareness, and prediction error) were based on Young, Davies [42] and Garfinkel, Seth [3].

*Heartbeat Counting Task (HCT)*. Participants were asked to count their heartbeat during 25s, 35s and 45s trials as used previously [43]. Order of the trials were randomised. A visual timer counted down from three to start each trial, then the word ‘START’ appeared. The screen then went blank, and participants were required to silently count their heartbeat until the word ‘STOP’ appeared on the screen. During each trial, R-R heartbeat intervals were recorded via a BIOPAC MP150 and ECG100C amplifier module (BIOPAC, USA). All participants were instructed only to report their felt heartbeats and not try to guess or use an exteroceptive aid (such as taking one’s pulse) [44]. After each trial, participants were required to disclose the number of counted heartbeats they felt. The length of each counting phase was not disclosed to the participants. At the end of each trial the participants immediately rated his/her confidence in their perceived accuracy of response. This confidence judgement was made using a computerised visual analogue scale with, 0 indicating “Not at all confident” and 100 “Completely confident”.

*Heartbeat Detection Task (HDT)*. A limitation of the heartbeat counting task is that participants can use knowledge about their heart rate to guide responses. The HDT overcomes this limitation and is therefore thought to provide a better measure of cardioceptive precision (i.e., sensitivity to the heartbeat) [45]. Unlike the HCT, the HDT has been shown to have good internal, construct, and face validity [35]. The task used in the present study consisted of three R-wave to stimulus intervals: (1) R + 0ms; (2) R + 200ms; and (3) random i.e., R + 100ms, 110ms, 125ms, 150ms, 200ms, 210 ms, 225ms or 250ms, shuffled randomly until all possibilities were used (i.e., on this type of trial stimuli were asynchronous with the heartbeat). R peaks were detected online by adjusting the gain on the ECG module so that the largest gain was applied without causing any clipping /distortion of the signal. This signal was then sent to DTU100 (Digital Trigger Unit), and the trigger output is input into the PC via the parallel port. This input informs the PC to immediately display the visual image on the screen for ~100ms. Participants viewed eight trials of each R-wave to stimulus interval in a random order [10]. Each trial consisted of a circle being presented on the screen for 60ms, and each trial consisted of eight circle presentations triggered by the participant’s heartbeat. The task required participants to judge whether heartbeat sensations are or are not simultaneous with the circle presentation. Participants indicated their response on the keyboard: 1 = in sync, 2 = not in sync. After each trial participants rated their confidence in their response on a single visual analogue scale (0—not at all confident, 100—completely confident). Overall, this task took approximately forty-five to sixty minutes to complete depending on resting heartrate and response speed.

#### Habitual exercise, depression, and anxiety

Depression / anxiety were measured as in Study 1. Exercise was measured by asking. participants ‘How many days per week do you take part in exercise of at least moderate intensity (i.e., enough to increase your heart rate / breathing rate) and for at least 30 minutes?’ This allowed us to quantify whether physical activities levels were consistent with WHO guidelines, and is in line with previous research [46].

### Data preparation

#### Interoceptive accuracy

For heartbeat counting accuracy the following transformation: 1 - ∑ (abs(Actual—Reported))/(Actual) was used to calculate heartbeat counting scores. These scores were then averaged to form a mean heartbeat counting score. The interoception score varied between 0 and 1 with a higher score indicating better accuracy.

It is generally accepted that on heartbeat detection tasks individuals are most likely to feel their heartbeat at ~ 200ms post R-peak [3]. Therefore, there are four possible outcomes on this task; HIT (responding YES to 200ms trials), MISS (responding NO to 200ms trials), FALSE ALARM (responding YES to random trials), CORRECT REJECTION (responding NO to random trials). 0ms trials served as a sensitivity analysis–that is, although on these trials’ stimuli are presented in synchrony with the heartbeat, detectors should be less able to detect their heartbeat this early in the cardiac cycle. The following scores were calculated:

1. percentage hits = number of hits / number of hits + number of misses;
2. percentage false alarms = number of false alarms / numbers of false alarms + number of correct rejections;
3. the previous scores can be contaminated by individual differences in response bias (i.e., different dominant tendencies to respond either by affirming or by denying that a stimulus was present), therefore, we also calculated a measure of overall performance accuracy across signal and non-signal trials: percentage hits–percentage false alarms / 2(percentage hits + percentage false alarms)–(percentage hits + percentage false alarms)^2^. This latter index was developed within the signal detection theory framework to provide a bias-free measure of overall performance [47]. Scores ranged from +1.0 to −1.0, with +1 indicating that all recorded responses were hits or correct rejections and −1 indicating all recorded responses were misses or false alarms (this was our primary outcome measure and results are reported below);
4. finally, it is plausible that athletes, by virtue of high levels of physical activity, may have developed a lower detection threshold, and thus be more likely to report feeling a heartbeat, irrespective of the nature of the trial. This propensity was indexed as the total number of YES responses across all trials;

For brevity results for percentage hit, percentage false alarms, and total number of YES responses are reported in S1 File.

#### Interoceptive metacognitive awareness, prediction error and bias

Heartbeat counting awareness was calculated using the within-participant Pearson correlation, r, between confidence and accuracy provided an index of interoceptive awareness.

Interoceptive prediction error was operationalized as the difference between objective interoceptive accuracy and subjective confidence [10]. Z scores were calculated for the interoceptive accuracy and confidence variables, and prediction values were calculated as the absolute difference between confidence and accuracy [10].

To establish the effects of metacognitive bias i.e., trait over / under confidence, a mean confidence score was calculated by averaging all confidence rating on the heartbeat counting task.

For the heartbeat detection task mean confidence for each type of outcome (MISS, FALSE ALARM, CORRECT REJECTION) was calculated.

### Statistical analysis

Data were analysed using SPSS 26 (IBM Corporation). As in Study 1, data were nested (Fig 2). Therefore, a series of hierarchical repeated measures ANOVAs were used–one for each interoceptive component which were entered as DVs. Crowd noise i.e., distraction (YES, NO) was the repeated measures factor. The rest of the analysis proceeded as in study 1, examining (1) the main effect of ATHLETE (i.e., athletes versus non-athletes), (2) EVENT (nested within ATHLETE) (i.e., sprinters versus distance athletes), (3) RANK (nested within ATHLETE) (i.e., elite (top 100) versus non-elite (not top 100) athletes), and (4) RANK X EVENT (nested within ATHLETE) (i.e., whether any effect of rank depended on the participants sport). Depression, anxiety, physical activity, and heartrate were entered as covariates. Where groups i.e., ATHLETE, EVENT or RANK, differed significantly regarding interoception, *t* tests were used to determine the nature of the effect (equal variances not assumed). Means (*SD*) for each group are reported in Table 3. With heartbeat detection confidence there was an additional repeated measures factor: outcome (HIT, MISS, FALSE ALARM, CORRECT REJECTION)–this allowed us to determine whether athletes varied in their overall confidence, and whether this confidence varied according to the accuracy of their response (insight). Cooks distance with a threshold of 4/N was used to identify potentially influential cases, and cases were excluded if they influenced the nature of the results–where relevant this is reported in the text. Bonferroni correction was used to control for the proportion of false positives.

### Results

#### Heartbeat Counting Task

**Fig 4:**
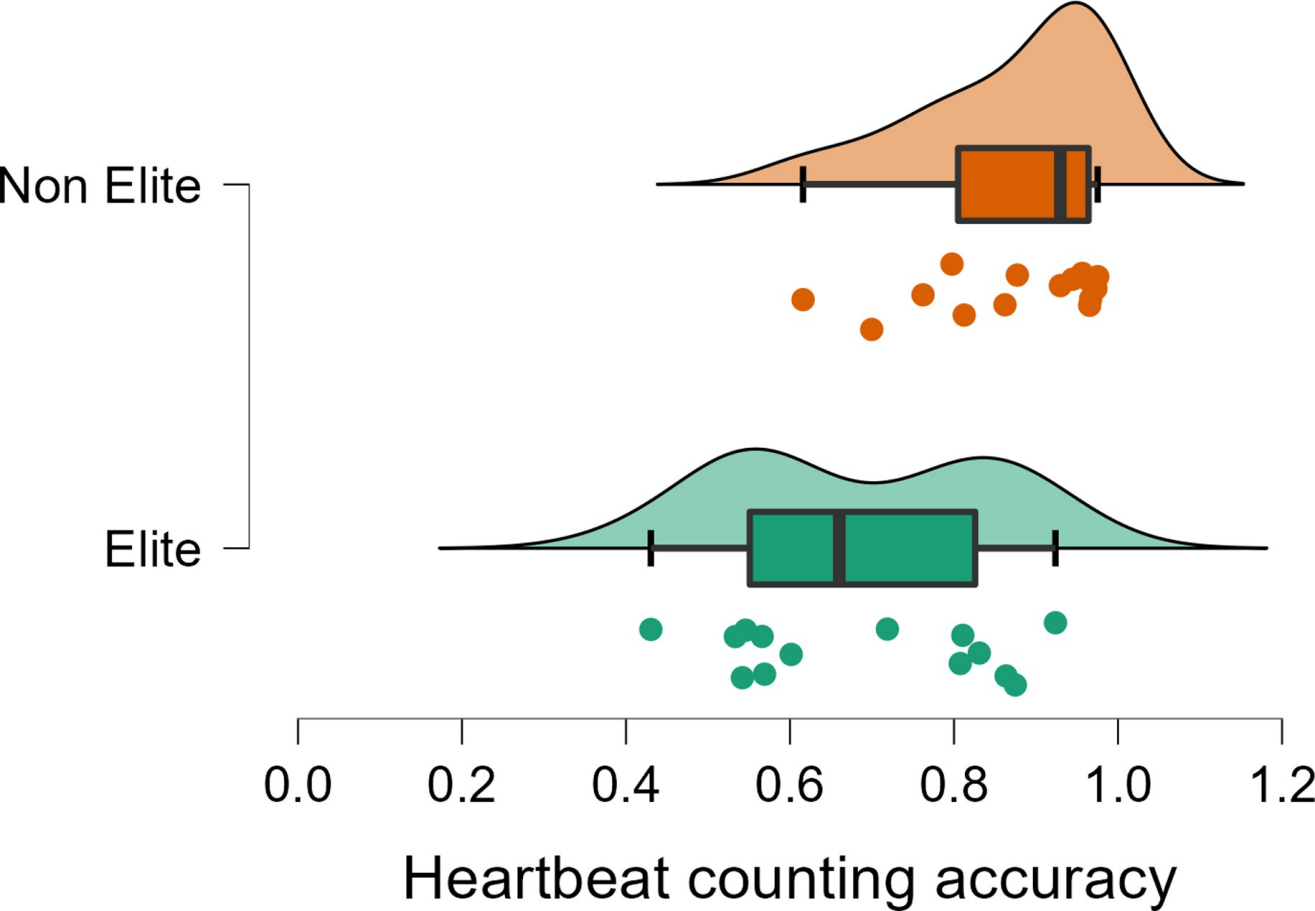
Raincloud plot showing heartbeat counting accuracy for elite and non-elite athletes. Note. Data are means after adjusting for depression, anxiety, habitual exercise, and HR.

*Interoceptive accuracy*. On no occasion was ATHLETE [ATHLETE X DISTRACTION (*F*(1, 49) = 0.35, *p* = .553, η^2^p = .007), ATHLETE (*F*(1, 49) = 0.27, *p* = .601, η^2^p = .006)], or EVENT [EVENT X DISTRACTION (*F*(1, 49) = 2.95, *p* = .092, η^2^p = .057), EVENT (*F*(1, 49) = 0.67, *p* = .414, η^2^p = .014)] related to heartbeat counting accuracy. The EVENT X RANK X DISTRACTION was also not significant (*F*(1, 49) = 0.00, *p* = .949, η^2^p = .000). However, there was a main effect of DISTRACTION (*F*(1, 49) = 6.24, *p* = .016, η^2^p = .113), and a significant DISTRACTION X RANK interaction (*F*(1, 49) = 4.77, *p* = .034, η^2^p = .089). *t* tests revealed that elite athletes performed more poorly than non-elite athletes when not distracted; *t*(23.09) = -3.57, *p* = .002. The effect was not significant during distraction; *t*(24.26) = -1.22, *p* = .234 (Fig 4).

*Interoceptive metacognitive awareness*. No significant effects were observed: DISTRACTION (*F*(1, 49) = 0.02, *p* = .874, η^2^p = .101); ATHLETE (*F*(1, 49) = 0.43, *p* = .836, η^2^p = .101); EVENT (*F*(1, 49) = 0.24, *p* = .621, η^2^p = .005); RANK (*F*(1, 49) = 0.45, *p* = .502, η^2^p = .009); DISTRACTION X ATHLETE (*F*(1, 49) = 0.01, *p* = .919, η^2^p = .000); DISTRACTION X EVENT (*F*(1, 49) = 0.02, *p* = .889, η^2^p = .000); DISTRACTION X RANK (*F*(1, 49) = 1.39, *p* = .244, η^2^p = .028); EVENT X RANK (*F*(1, 49) = 0.63, *p* = .428, η^2^p = .013); EVENT X RANK X DISTRACTION (*F*(1, 49) = 0.23, *p* = .629, η^2^p = .005).

*Interoceptive confidence*. Although the ATHLETE X DISTRACTION interaction was not significant (*F*(1, 49) = 1.03, *p* = .314, η^2^p = .021), there was a main effect of ATHLETE (*F*(1, 49) = 7.38, *p* = .009, η^2^p = .131); athletes were more confident than non-athletes. No other significant effects were observed: No significant effects were observed: DISTRACTION (*F*(1, 49) = 0.001, *p* = .971, η^2^p = .000); EVENT (*F*(1, 48) = 0.25, *p* = .614, η^2^p = .005); RANK (*F*(1, 49) = 0.77, *p* = .383, η^2^p = .016); DISTRACTION X EVENT (*F*(1, 49) = 0.47, *p* = .495, η^2^p = .010); DISTRACTION X RANK (*F*(1, 49) = 0.00, *p* = .924, η^2^p = .000); EVENT X RANK (*F*(1, 49) = 0.26, *p* = .872, η^2^p = .001); EVENT X RANK X DISTRACTION (*F*(1, 49) = 0.97, *p* = .328, η^2^p = .020).

*Prediction error*. The effect of DISTRACTION was significant (*F*(1, 49) = 4.46, *p* = .040, η^2^p = .084). However, on no occasion was ATHLETE [ATHLETE X DISTRACTION (*F*(1, 49) = 0.66, *p* = .420, η^2^p = .013), ATHLETE (*F*(1, 49) = 0.10, *p* = .923, η^2^p = .000)], or EVENT [EVENT X DISTRACTION (*F*(1, 49) = 1.08, *p* = .303, η^2^p = .022), EVENT (*F*(1, 49) = 1.64, *p* = .206, η^2^p = .032)] related to prediction error. Although the DISTRACTION X RANK (*F*(1, 49) = 1.57, *p* = .216, η^2^p = .014), the EVENT X RANK (*F*(1, 49) = 0.203, *p* = .655, η^2^p = .004) and the EVENT X RANK X DISTRACTION (*F*(1, 49) = 0.06, *p* = .800, η^2^p = .001) interactions were not significant, there was a tendency for elite athletes to have a higher prediction error: RANK (*F*(1, 49) = 3.92, *p* = .053, η^2^p = .074). This latter effect did not survive the FDR correction and so should be interpreted cautiously.

#### Heartbeat Detection Task

**Fig 5:**
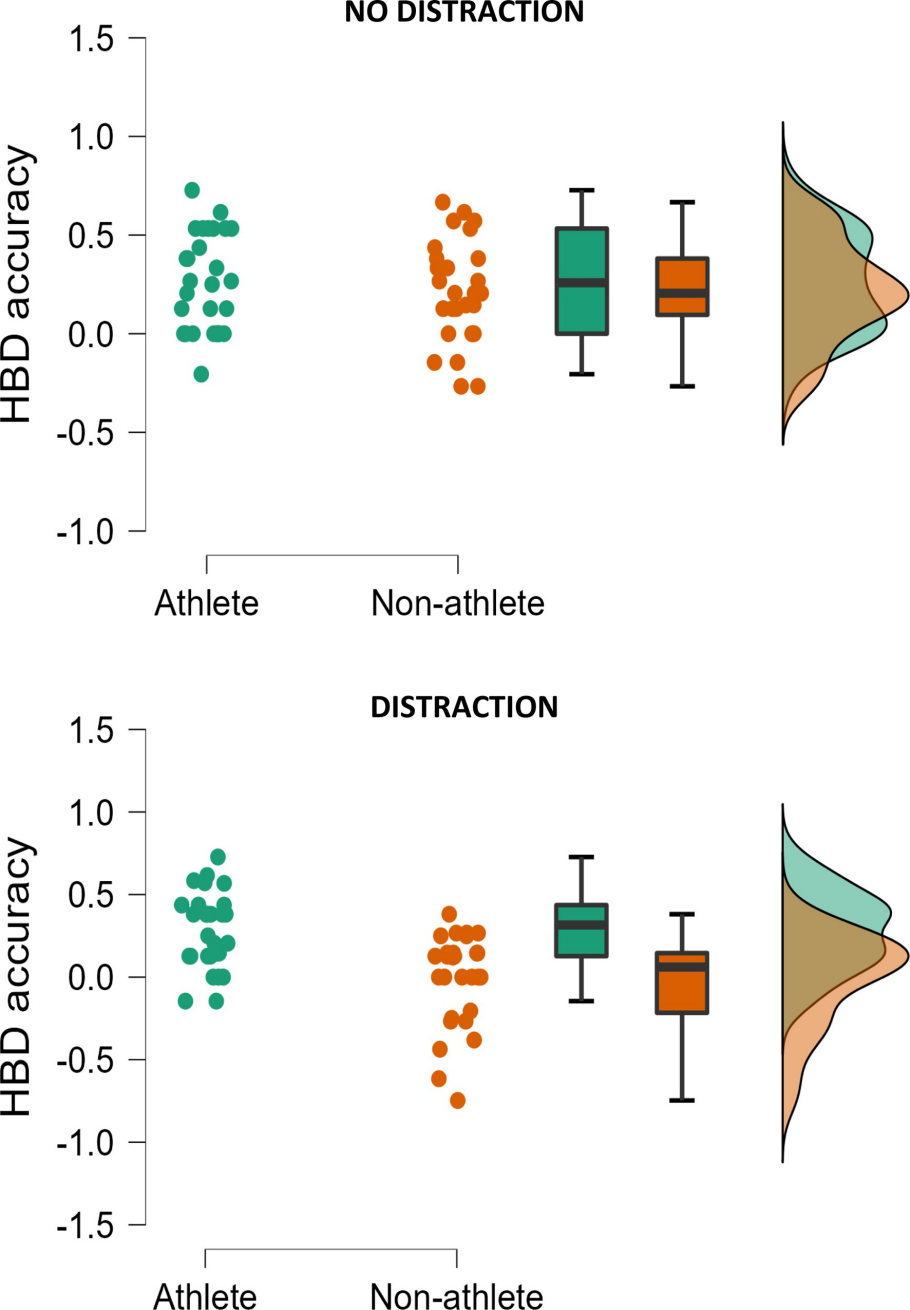
Raincloud plot showing heartbeat detection accuracy in athletes and non-athletes. Note. Data are means after adjusting for depression, anxiety, habitual exercise, and HR. HBD–heartbeat detection.

*Accuracy score*. The effect of DISTRACTION was not significant (*F*(1, 47) = 0.47, *p* = .496, η^2^p = .010). Similarly, there was no effect of RANK (*F*(1, 47) = 0.06, *p* = .940, η^2^p = .000), nor a RANK X DISTRACTION interaction (*F*(1, 47) = 1.15, *p* = .289, η^2^p = .024). In addition, the RANK X EVENT X DISTRACTION interaction did not reach significance (*F*(1, 47) = 0.32, *p* = .571, η^2^p = .007), and neither did the DISTRACTION X EVENT interaction (*F*(1, 47) = 0.27, *p* = .606, η^2^p = .006). However, there was a significant main effect of ATHLETE (*F*(1, 49) = 11.93, *p* = .001, η^2^p = .203), but this was superseded by a significant DISTRACTION X ATHLETE interaction (*F*(1, 49) = 4.49, *p* = .039, η^2^p = .087). *t* tests revealed that athletes performed more accurately than non-athletes when distracted; *t*(53.37) = 4.07, *p* < .001. The effect was not significant when participants were not distracted; *t*(53.97) = 0.64, *p* = .522 (Fig 5).

*Insight and confidence*. Two outliers with a Cook’s distance > 0.07 were identified and removed. The TRIAL X RESPONSE interaction was significant (*F*(1, 43) = 4.19, *p* = .046, η^2^p = .076), indicating that overall participants had insight into the accuracy of their responses. However, the TRIAL X RESPONSE X DISTRACTION interaction was not significant (*F*(1, 43) = 0.03, *p* = .863, η^2^p = .001), indicating that participants confidence did not depend on the level of distraction.

Insight did not vary depending on ATHLETE [ATHLETE X TRIAL X RESPONSE (*F*(1, 43) = 1.60, *p* = .212, η^2^p = .036), ATHLETE X TRIAL X RESPONSE X DISTRACTION (*F*(1, 43) = 0.68, *p* = .412, η^2^p = .016), EVENT [EVENT X TRIAL X RESPONSE (*F*(1, 43) = 0.56, *p* = .456, η^2^p = .013), EVENT X TRIAL X RESPONSE X DISTRACTION (*F*(1, 43) = 2.29, *p* = .137, η^2^p = .051), or RANK [RANK X TRIAL X RESPONSE (*F*(1, 43) = 0.24, *p* = .627, η^2^p = .006), RANK X TRIAL X RESPONSE X DISTRACTION (*F*(1, 43) = 0.36, *p* = .550, η^2^p = .008).

Similarly, the main effects of EVENT (*F*(1, 43) = 0.00, *p* = .960, η^2^p = .000), RANK (*F*(1, 43) = 0.15, *p* = .693, η^2^p = .004) and the RANK X EVENT interaction (*F*(1, 43) = 0.68, *p* = .201, η^2^p = .038) were not significant. However, the main effect of ATHLETE was significant (*F*(1, 43) = 5.98, *p* = .019, η^2^p = .122); overall athletes were more confident than non-athletes.

### Discussion and theoretical integration

The aim of the present studies was to examine interoceptive differences in elite sprint and long-distance runners. Key findings are summarised in Table 4. These included: (I) compared to non-athletes, athletes (i.e., both sprinters and distance runners) had more confidence (Study 2) in their interoceptive percept, and reported trusting their body more, using it to self-regulate and having better attentional control towards their body (Study 1); (II) sprinters reported having better regulation of attention to internal sensations, more emotional awareness, better self-regulation, and a greater propensity to listen to their body for insight than both distance runners (Fig 3), (III) athletes (i.e., both sprinters and distance runners) were better able to maintain heartbeat detection performance when distracted compared to non-athletes (Fig 5), and (IV) elite athletes were characterised by lower emotional awareness, self-regulation, and body listening (Study 1), and were less accurate at counting their heartbeat when not distracted (Fig 4), and characterised by a higher interoceptive prediction error (Study 2). The present pattern of results supported the view that athletic populations have altered interoceptive abilities, including higher interoceptive confidence and a better ability to maintain attention to internal sensations when distracted. However, contrary to expectations elite athletes did not have better interoceptive abilities.

**Table 4:** Results from Study 1 and Study 2 which examined the association between interoception, type of sport and level of athlete.

	Athlete	Event	Rank
**MAIA Noticing**	ns	ns	ns
**MAIA Attention regulation**	Athl. > Non.	Sprint > Dist.	ns
**MAIA Emotional Awareness**	ns	Sprint > Dist.	Lower in elite
**MAIA Self-Regulation**	Athl. > Non.	Sprint > Dist.	Lower in elite
**MAIA Body Listening**	ns	Sprint > Dist.	Lower in elite
**MAIA Trusting**	Athl. > Non.	ns	ns
**Heartbeat counting accuracy**	ns	ns	Lower in elite when not distracted
**Heartbeat counting awareness**	ns	ns	ns
**Heartbeat counting prediction error**	ns	ns	Higher in elite
**Heartbeat counting confidence**	Athl. > Non.	ns	ns
**Heartbeat detection accuracy**	Athl. > Non. when distracted	ns	ns
**Heartbeat detection confidence**	Athl. > Non.	ns	ns
**Heartbeat detection insight**	ns	ns	ns

*Note*: Sprint—sprinter, Dist.—distance runner, Non.—non-athlete, Athl.–athlete.

#### Event

*Self-reported interoception*. In Study 1, self-reported interoceptive sensibility varied by both type of sport and level of the athlete (Fig 3). On four out of six of the MAIA subscales analysed here, sprinters reported having better interoceptive abilities than both distance runners (Table 4). To the best of our knowledge only one other study has compared long-distance and sprint athletes using the MAIA [32]. It was found that sprinters reported higher attention control to their own bodies than did long-distance runners [32]. The present results supported this observation and suggested that sprint athletes may believe that they have a heightened ability to control and regulate attention to bodily signals. However, Hirao, Vogt [32] did not find that athletes differed on any other MAIA dimension. Given the relatively small sample used by Hirao, Vogt [32] (17 long-distance runners and 15 sprinters), compared to that of the present study (49 sprinters and 62 distance runners), Hirao, Vogt [32] may have lacked the power to detect effects on the other MAIA dimensions. Indeed, here observed effect sizes were small to medium. In addition, on several occasions in the present study the strongest effects occurred when athletes, irrespective of their sport, were compared with non-athletes. As Hirao, Vogt [32] did not include non-athletes this may explain their failure to observe significant effects.

**Fig 6:**
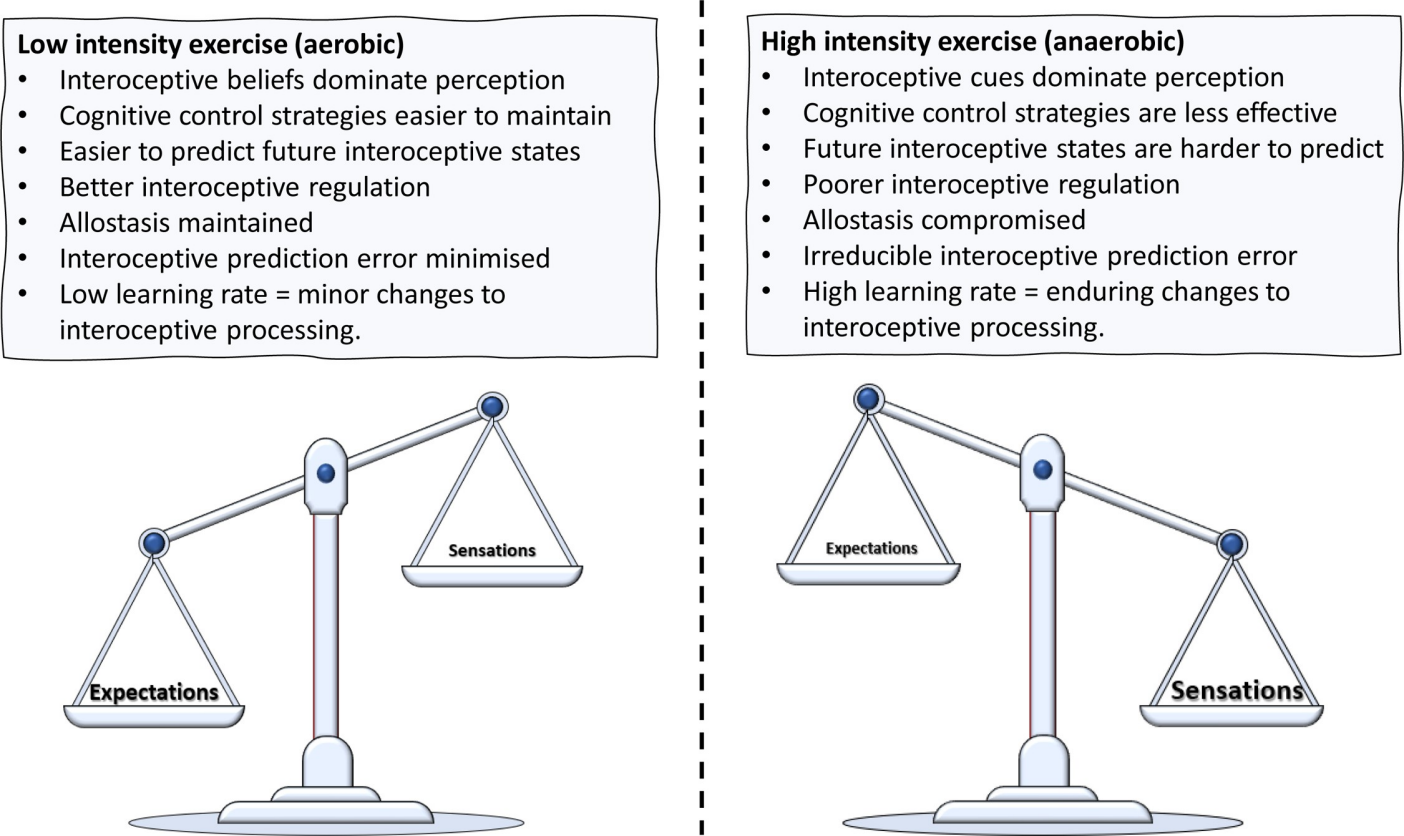
Interoceptive processing in response to high and low intensity exercise. Note. This model shows the role of exercise intensity in modulating the interaction between ‘top-down’ and ‘bottom-up’ interoceptive processes. During high intensity exercise interoceptive signals dominate perception such that it becomes more difficult to engage ‘top-down’ cognitive resources and predict future interoceptive states. Conversely, when exercise intensity is low, ‘top-down’ cognitive strategies are easier to maintain and future interoceptive states are easier to predict.

As recent models of physical activity and interoception have emphasised that exercise intensity modulates the interaction between cognitive and interoceptive factors [17–20], it is interesting that here, it was often sprint athletes, not distance runners, who reported superior interoceptive abilities (Fig 3 and Table 4). For example, previously, it was argued that ‘top-down’ cognitive strategies (such as associative / dissociative attention [48, 49]) are usually most effective at low to moderate intensities [18, 50]. At maximal intensities ‘bottom-up’ anaerobic interoceptive cues are more salient making the use of cognitive strategies more challenging [18]. Similarly, McMorris [20] suggested that at maximal intensities, it becomes difficult to produce a ‘top-down’ interoceptive prediction based on past experience (Fig 6). Notably, empirical research indicated some athletes may be relatively immune to these decremental effects of high intensity exercise [51]. Thus, the present finding, that sprint athletes, who regularly train at or above their anaerobic threshold, report having better interoception, could reflect a superior ability to control ‘top-down’ attention to internal states. As we did not assess interoception during exercise of different intensities, this will be important for future research. It should also be considered whether these are innate characteristics of sprint athletes, or a consequence of their participation in high intensity exercise. For example, given the dominance of interoceptive cues at high intensities (e.g., during sprints), it makes sense that high intensity exercise may be most efficacious in producing enduring changes in interoceptive processing.

*Interoceptive accuracy*. That said, when we assessed interoception using behavioural tests (Study 2) findings indicated that athletes had better interoception irrespective of their sport or regular training intensity. Specifically, athletes (i.e., *both* sprint and distance athletes) were better able to perform the heartbeat detection task when distracted. Non-athletes had a significant decline in heartbeat detection accuracy when exposed to crowd noise (distraction); an effect not observed in either type of athlete (Fig 5). Whist these findings might indicate that intensity is less important than previously stated [17–20], we did not systematically manipulate training intensity, and whilst distance runners may spend a greater amount of time training in their aerobic zone, anaerobic interval training will still form part of their regular training routine. It is also possible that at high training loads, such as those seen in competitive athletes, there could be a ceiling effect due to the strength of the effect of physical activity in general. This could eliminate any differences between sports. Consequently, experimental research is needed to confirm a link between training intensity in interoception.

There are also several plausible explanations for better interoceptive abilities in athletes that require further exploration. For instance, previously it was reported that in children greater activity levels and physical fitness were positively correlated with interoceptive accuracy [24]. Therefore, one possibility is that the present observations may be mediated by athletes’ superior fitness levels. However, when resting heart rate was included in the analysis (an indicator of general fitness [52]) the results remained the same. Although replication using a wider array of physical fitness indices (e.g., V02 max, echocardiogram to assess structural heart changes, baroreceptor sensitivity) is needed, the present findings suggested that additional mechanisms besides physical fitness might be involved.

Another possibility is that an increased salience of interoceptive afferents during and after physical activity promotes the development of more accurate internal interoceptive models (Fig 1). Indeed, there is evidence that both acute and chronic physical activity can enhance interoceptive abilities. For example, Jones and Hollandsworth [31] reported improvements in interoceptive accuracy after exercise that raised resting heartrate by 75%. In addition, a twelve-week physical activity intervention improved interoception in previously sedentary individuals, albeit in an appetitive rather than a cardioceptive domain [53]. The mechanisms mediating these interoceptive adaptations are currently unknown but might include general increases in attentional capacity, changes in dopaminergic and / or oxytocinergic activity, neuroplasticity, or physiological remodelling [17, 53].

Interestingly, once developed, accurate interoceptive representations are said to contribute to maintaining allostasis in response to subsequent physiological perturbations [22, 23, 25, 30], resulting in a positive feedback loop (Fig 1). Theoretically, having more accurate internal models could also facilitate interoceptive accuracy during distraction, when the ability to attend to ‘bottom-up’ sensory input is compromised (Fig 1). To the extent that accurate interoception facilitates performance (see later), the ability to maintain interoceptive accuracy during distraction could have ramifications for competition when distractions such as crowd noise are common. Although we statistically controlled for current activity levels it is likely that athletes will have accumulated a larger volume of training affording many more opportunities for interoceptive learning, compared to non-athletes. Future research which captures a more comprehensive training history of participants might be profitable.

*Confidence and insight*. A final observation was that here, athletes were more confident in their interoceptive percept (Study 2). Note that effects did not vary by type of athlete. This indicated that irrespective of their sport, athletes repeated exposure to increased interoceptive signal transmission during physical training may overtime increase interoceptive confidence. Interestingly, on no occasion did interoceptive confidence depend on the level of distraction. This suggested that participants confidence ratings may have been based on their prior interoceptive experiences rather than their current afferent inputs. Further experimental research that assesses confidence both prospectively and retrospectively, in naive and experienced athletes, may be able to confirm these interpretations.

It is notable that on the heartbeat detection task, confidence varied depending on whether participants gave a correct or an incorrect response. This suggested that in general participants were able to discriminate the accuracy of their interoceptive percept. However, this latter effect was not dependant on whether participants were athletes. Therefore, the increased confidence in athletes could in fact reflect a metacognitive bias (i.e., the propensity to report overconfidence) rather than a better ability to track their correct and incorrect responses. Indeed, this interpretation is supported by the observation that in the present study athletes and non-athletes did not differ in their heartbeat counting metacognitive awareness, although the limitations of this latter task should be considered [10, 23, 30].

#### Level of athlete

*Self-reported interoception*. When the effects of rank were considered in Study 1, elite athletes reported lower levels of emotional awareness, self-regulation, and body listening than their more novice counterparts; effects that were confined to sprint athletes. This was unexpected but, contrary to previous suggestions [32], it indicated that a high body awareness could interfere with the performance of elite athletes. Indeed, when it comes to the control of movement, it is recognised that explicit awareness can disrupt the execution of movements and hinder performance in elite athletes [54]. For example, Ille, Selin [55] found that sprint start reaction times, and 10-meter sprint times, were significantly shorter when athletes received external focus instructions, than when they received internal focus instructions. Hitherto, it is not known whether similar principles apply to interoception. However, it is plausible that an excessive ‘awareness of the connection between body sensations and emotional states’, or a high propensity to ‘listen to the body for insight’ could hinder elite performance e.g., by increasing perceptions of fatigue. As such, until firm conclusions can be drawn from future research, the present results indicated that some mindfulness-based interventions which emphasise a focus on the body (e.g., body scan techniques) should be used with caution.

*Interoceptive accuracy*. Findings from Study 2 supported those in Study 1: elite athletes less accurately counted their heartbeat (when not distracted) and were characterised by a higher interoceptive prediction error. One interpretation is that during training and competition elite athletes may routinely push past their natural physiological boundaries in the pursuit of performance excellence. Doing so may require more weight be afforded to prior predictions so that afferent bodily sensations that signal the onset of fatigue can be ignored. Overtime, this tendency may contribute to a ‘mismatch’ between expected and actual bodily states reducing interoceptive accuracy. If correct, the psychological and physiological consequences of this situation are potentially serious and will need to be examined. It is important to consider that in the present research interoception was measured at rest. It is possible that interoceptive advantages only become evident under conditions of physiological arousal. This should be explored in future research.

However, given the small sample size in Study 2, and the fact that no effects based on rank were observed using the heartbeat detection task, more data are required before firm conclusions can be drawn. For example, heartbeat counting performance is known to be influenced by explicit knowledge of heartrate [45]. Athletic populations are more likely to use a range of sports technologies that provide interoceptive feedback through exteroceptive channels (e.g., heartrate monitors). Therefore, the possibility that expert differences are due to differences in knowledge about heartrate needs to be excluded. Additionally, it is plausible that a heavy use of exteroceptive aids could in fact reduce awareness of internal states. Nonetheless, whilst the accuracy of heartrate knowledge may bias the athlete versus non-athlete comparison, it seems less likely to have influenced our findings based on the level of the athlete.

#### General limitations

Other limitations need to be considered. For example, in both Study 1 and Study 2 the cross-sectional design of this research means that we cannot determine whether interoceptive differences between athletes and non-athletes are innate or a product of their training. Future longitudinal research might be able to determine this. An important question is how athletes should be classified based on their sport. For example, here 800m runners were classified as distance runners. However, their sports would tend to involve some sprint training although mostly aerobic work. Removing these cases from our group did not alter the present results, however, future research should be mindful of how best to classify sprint and distance athletes. Given the limited availability of elite athletes, sample size in our lab-based study (Study 2) is an inevitable concern especially concerning the three-way interaction [56]. Although we were able to overcome this in Study 1 by conducting the study online this limited us to survey methods which are known to only weakly correlate with objective interoception methods [10, 30]. Future research may be able to better deal with this issue by using newly developed interoception tasks such as the Phase Adjustment Task which can be run remotely [57]. It is also worth considering that the distractor task might be idiosyncratic to athletes. Therefore, future research replicating these findings using other kinds of distractors might prove profitable. It might also be interesting to determine whether the effects observed here using the MAIA replicate using other more recently developed interoception scales such as the Interoceptive Accuracy Scale [4] and the Interoceptive Attention Scales [58]. Physical activity levels were only available for Study 2, therefore, future work replicating Study 1 while controlling for activity levels are required. In addition, Study 2 included a male only sample so the effects should be replicated in females. Here we focused on cardioception. However, it is possible that effects may differ in other domains such as respiration. Future research might delineate which of these two interoceptive domains are most meaningful for athletic performance. Due to limitations of the of the heartbeat detection task we were unable to calculate the more robust meta ‘*d’ or* ROC AUC measure of metacognition. Future research using a larger number of trials might benefit from using these measures. Finally, physically active populations may also differ in terms of nutrition, and in the future, it should be considered whether this contributed to the present results.

## Conclusions

We observed a range of interoceptive differences in world-class sprint and long-distance runners. Sprinters reported more body listening, better self-regulation, and higher levels of interoceptive attentional control, and better emotional awareness (Study 1). Compared to non-athletes, athletes regardless of their sport, had more confidence in their interoceptive percept (Study 2), trusted their body more (Study 1), and were better able to maintain interoceptive accuracy while distracted by crowd noise (Study 2). We propose a positive feedback cycle whereby exposure to salient afferents during physical exercise facilitates the development of accurate predictive models, which in turn contribute to maintaining allostasis in response to subsequent physiological and psychological perturbations (Fig 5). Surprisingly, elite athletes were characterised by lower emotional awareness, self-regulation, and body listening (Study 1), and were less able to count their heartbeat, and characterised by a higher interoceptive prediction error (Study 2). Contrary to previous suggestions, the present data indicated that elite athletes may be characterised by lower awareness of their internal states. Future research should consider whether training beyond one’s physiological boundaries requires the excessive attenuation of interoceptive states that overtime contributes to an enduring ‘mismatch’ between expected and actual bodily states.

## Supporting information

S1 File This file contains the results for percentage hit, percentage false alarms, and total number of YES responses. (DOCX)
