# Supplementary material for: Interoceptive differences in elite sprint and long-distance runners: A multidimensional investigation
Source: PLoS One. 2023 Jan 25;18(1):e0278067. doi: 10.1371/journal.pone.0278067 (PMC9876362; doi:10.1371/journal.pone.0278067)
Supplement: S1 File — (DOCX) [file pone.0278067.s001.docx]

**Supplementary information (S1)**

Hit rate

The effect of DISTRACTION was not significant (*F*(1, 49) = 0.19, *p* = .657, η^2^p = .004). Similarly, on no occasion was RANK [RANK X DISTRACTION (*F*(1, 49) = 3.04, *p* = .087, η^2^p = .059), RANK (*F*(1, 49) = 0.12, *p* = .727, η^2^p = .003)], or EVENT [EVENT X DISTRACTION (*F*(1, 49) = 0.26, *p* = .607, η^2^p = .005), EVENT (*F*(1, 49) = 2.66, *p* = .109, η^2^p = .052), EVENT X RANK X DISTRACTION (*F*(1, 49) = 0.02, *p* = .966, η^2^p = .000)] related to hit rate. Similarly, the DISTRACTION X ATHLETE interaction was not significant (*F*(1, 49) = 1.51, *p* = .224, η^2^p = .030). However, there was a main effect of ATHLETE (*F*(1, 49) = 6.69, *p* = .013, η^2^p = .120); athletes had a higher hit rate than non-athletes.

False alarms

No significant effects were observed: DISTRACTION (*F*(1, 49) = 2.46, *p* = .123, η^2^p = .048); ATHLETE (*F*(1, 49) = 0.00, *p* = .966, η^2^p = .000); EVENT (*F*(1, 49) = 0.19, *p* = .890, η^2^p = .000); RANK (*F*(1, 49) = 0.50, *p* = .480, η^2^p = .010); DISTRACTION X ATHLETE (*F*(1, 49) = 0.77, *p* = .383, η^2^p = .016); DISTRACTION X EVENT (*F*(1, 49) = 0.93, *p* = .762, η^2^p = .002); DISTRACTION X RANK (*F*(1, 49) = 0.69, *p* = .408, η^2^p = .014); EVENT X RANK (*F*(1, 49) = 0.34, *p* = .562, η^2^p = .007); EVENT X RANK X DISTRACTION (*F*(1, 49) = 0.13, *p* = .715, η^2^p = .003).

Sensitivity analysis (hit rate on zero millisecond trials)

No significant effects were observed: DISTRACTION (*F*(1, 49) = 0.12, *p* = .727, η^2^p = .003); ATHLETE (*F*(1, 49) = 0.17, *p* = .678, η^2^p = .004); EVENT (*F*(1, 49) = 0.618, *p* = .436, η^2^p = .012); RANK (*F*(1, 49) = 0.16, *p* = .683, η^2^p = .003); DISTRACTION X ATHLETE (*F*(1, 49) = 0.22, *p* = .638, η^2^p = .005); DISTRACTION X EVENT (*F*(1, 49) = 0.77, *p* = .384, η^2^p = .016); DISTRACTION X RANK (*F*(1, 49) = 0.25, *p* = .618, η^2^p = .005); EVENT X RANK (*F*(1, 49) = 0.16, *p* = .683, η^2^p = .003); EVENT X RANK X DISTRACTION (*F*(1, 49) = 0.06, *p* = .802, η^2^p = .001).

Number of YES responses

Again, no significant effects were observed: DISTRACTION (*F*(1, 49) = 0.96, *p* = .331, η^2^p = .019); ATHLETE (*F*(1, 49) = 1.10, *p* = .298, η^2^p = .022); EVENT (*F*(1, 49) = 0.67, *p* = .414, η^2^p = .014); RANK (*F*(1, 49) = 0.22, *p* = .640, η^2^p = .004); DISTRACTION X ATHLETE (*F*(1, 49) = 0.00, *p* = .960, η^2^p = .000); DISTRACTION X EVENT (*F*(1, 49) = 0.87, *p* = .354, η^2^p = .018); DISTRACTION X RANK (*F*(1, 49) = 1.49, *p* = .227, η^2^p = .030); EVENT X RANK (*F*(1, 49) = 0.22, *p* = .640, η^2^p = .004); EVENT X RANK X DISTRACTION (*F*(1, 49) = 0.03, *p* = .956, η^2^p = .000).
